# Multimodal Patient-Specific Registration for Breast Imaging Using Biomechanical Modeling with Reference to AI Evaluation of Breast Tumor Change

**DOI:** 10.3390/life11080747

**Published:** 2021-07-26

**Authors:** Cheng Xue, Fuk-Hay Tang, Christopher W. K. Lai, Lars J. Grimm, Joseph Y. Lo

**Affiliations:** 1 School of Medical and Health Sciences, Tung Wah College, Hong Kong, China; cxue@twc.edu.hk; 2 Health and Social Sciences, Singapore Institute of Technology, Singapore 138683, Singapore; chris.lai@singaporetech.edu.sg; 3 Carl E. Ravin Advanced Imaging Laboratories, Department of Radiology, Duke University Medical Center, Durham, NC 27705, USA; lars.grimm@duke.edu (L.J.G.); joseph.lo@duke.edu (J.Y.L.)

**Keywords:** biomechanics, medical image registration, breast cancer, finite element method, patient specific

## Abstract

Background: The strategy to combat the problem associated with large deformations in the breast due to the difference in the medical imaging of patient posture plays a vital role in multimodal medical image registration with artificial intelligence (AI) initiatives. How to build a breast biomechanical model simulating the large-scale deformation of soft tissue remains a challenge but is highly desirable. Methods: This study proposed a hybrid individual-specific registration model of the breast combining finite element analysis, property optimization, and affine transformation to register breast images. During the registration process, the mechanical properties of the breast tissues were individually assigned using an optimization process, which allowed the model to become patient specific. Evaluation and results: The proposed method has been extensively tested on two datasets collected from two independent institutions, one from America and another from Hong Kong. Conclusions: Our method can accurately predict the deformation of breasts from the supine to prone position for both the Hong Kong and American samples, with a small target registration error of lesions.

## 1. Introduction

**Figure 1:**
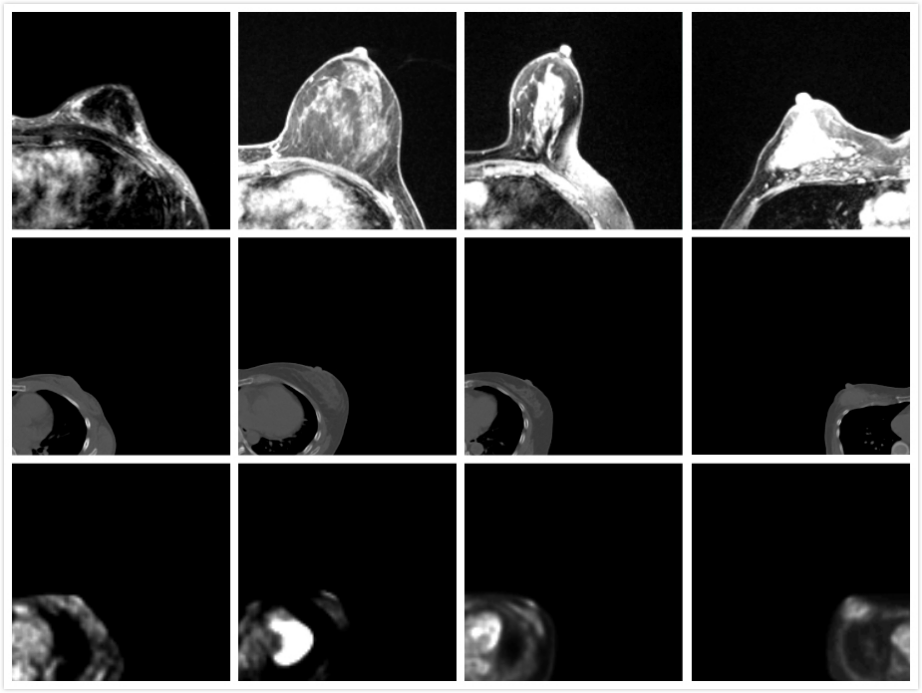
Appearance of MRI (**first row**), CT (**second row**), and PET images (**third row**). The direction of MRI is adjusted.

Breast cancer is the second most prevalent type of death-causing cancer in women worldwide [1]. It is therefore important to screen women for early detection of breast cancer, especially using computer-aided detection or artificial intelligence (AI) technology. In clinical settings, registered positron emission tomography (PET) and magnetic resonance imaging (MRI) images provide both structural and functional information, which can enhance the information of the medical image and help clinicians make a diagnosis. This further paves the way for development of AI detection and evaluation of cancer changes, such as temporal subtraction where the changes can be detected at different time intervals. Previous research also showed the superior performance of AI algorithms [2,3,4]. The registration of PET and MRI remains challenging for three reasons: (1) the patient position is different during PET and MR scanning. The patient is in a supine position during PET scanning and prone position in MR scanning. (2) Deformation of the breast is large. It is commonly known that the breast contains different types of soft tissues, such as adipose and fibro-glandular tissues; this anatomy allows the breast to easily deform. (3) The low resolution of PET images makes it difficult for them to be registered, as shown in Figure 1. Typically, preoperative diagnostic MR breast images are obtained with the patient in the prone position because the breasts are flattened and suboptimally visualized in the supine position, although it is still possible to acquire supine MR images, but a dedicated supine breast coil is needed to maintain the MR image quality [5]. Several studies have proved that prone breast PET images can improve the detection of breast cancer [6,7,8], but a positioning device is required. Therefore, the registration of PET and MR in the prone position is still a promising topic. Therefore, we propose to utilize a biomechanical model to simulate the deformation of the breast from the supine to prone position, and further finish registration of PET and MR images. It has the potential to assist in the diagnosis and treatment of breast cancer.

Finite element modeling (FEM) is one of the most popular physical simulations in the field of engineering, such as mechanical engineering and biomechanics engineering [9,10]. By assigning reasonable tissue property and boundary conditions, finite element analysis can provide realistic deformation predictions. Biomechanical models have been used to simulate the deformation of the lung [11] and the breast such as compression in X-ray mammography [12,13], and behavior under gravity from prone to supine positions [5,14,15]. Han et al. developed a homogeneous model to predict large breast deformations from the prone position to supine position [15], but the mechanical property variance has not been considered. In this regard, a novel biomechanical model is proposed in this study. Concretely, the finite element method is adopted to simulate the breast deformation from a supine position to a prone position. The mechanical property variance and reference state recognition are considered, which are important for patient-specific modeling. Furthermore, due to the lacking of realistic material property data, a mechanical property optimization process is proposed to build a patient-specific model. In this way, the variations in the mechanical properties of soft tissues are considered. The deformation is also refined by an affine transformation to further improve the performance. Our model was tested on two positron emission tomography (PET)/computed tomography (CT) and MR datasets collected from two independent medical institutions in Hong Kong and America. The breast size of Asian and Caucasian women is usually different due to the different diets and genetic variants, fat/glandular tissue composition, and contouring of the breast. Normally, Asian women have smaller, denser breasts than Caucasian women [16]. It is challenging to build a general model for patients with a wide range of breast sizes or densities.

The main contributions of the study are:

- A biomechanical model-based non-rigid image registration method that provides physically realistic deformation estimations is proposed;
- A mechanical property optimization process for various physical properties is proposed to make the model patient specific;
- Our model was tested on datasets collected from two independent institutions in Hong Kong and America.

## 2. Method

### 2.1. Dataset

**Figure 2:**
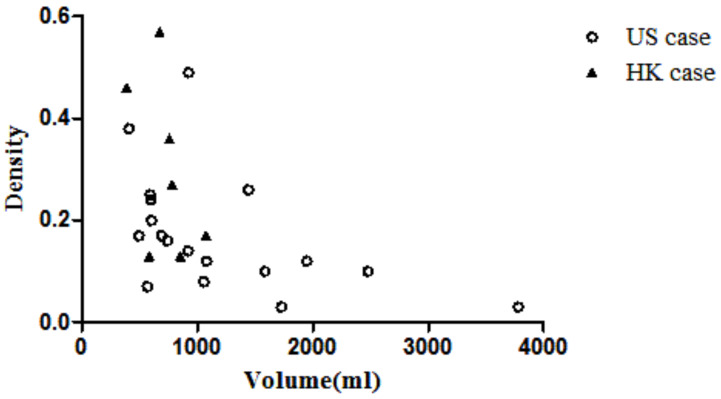
Scatter plot of volume vs. density.

To ensure a wide range of breast densities and volumes, we collected 20 PET/CT and matching MR image sets from the Duke University Health System in the Unite States (GE Discovery STE and Siemens Avanto) and 8 PET/CT and MR images from Hong Kong Sanatorium Hospital (Siemens Biograph 40 and Siemens Trio TIM). The scans were acquired within 6 months of each other as part of routine clinical practice. Two pairs of images (US19 and US20) were excluded from the former as the breasts were in contact with the MR coil. Thus, the deformation of the breast is not only a result of gravity but also the holding force from the coil, which is beyond the scope of this study. One pair of breast images from the Hong Kong sample (HK5) was excluded because there was no clear cancer lesion in the MR image. Therefore, a total of 25 image sets (both PET/CT and MR) were used in this study. The MR image resolution was 1.3 pixels per mm and the PET image resolution was around 0.26 pixels per mm in this study. Since the large difference between the PET and MR images could affect the accuracy of image registration, all the images were interpolated linearly to a resolution of 1 pixel per mm. The breast volume and density of samples from the two datasets are plotted in Figure 2. It shows that Asian women have denser breasts than US women.

Table 1 shows the comparison between American samples and Hong Kong samples. The median volume and median volumetric density are 990 mL and 0.15 for the American samples and 762 mL and 0.27 for the Hong Kong samples. The density of the American samples is significantly lower than that of the Hong Kong samples (*p* < 0.05), but there is no difference between the volumes. The volmetric density is defined as the volume of the fibro-glandular tissue divided by the volume of the breast region.

### 2.2. Modeling

**Figure 3:**
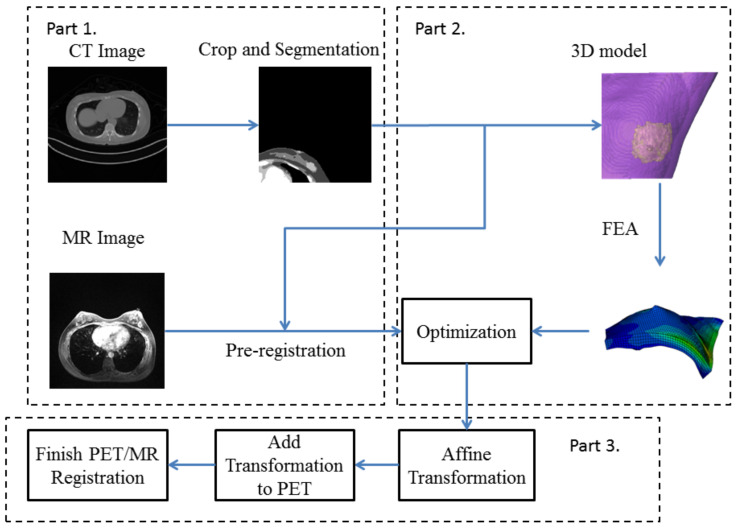
Flowchart of our proposed deformable registration model. Part 1 is the pre-processing step. Part 2 includes the finite element analysis (FEA) and optimization. Part 3 transforms the coordinate of step 2 to PET image to generate transformed PET images and final registered PET/MR images.

The framework of our method can be divided into three parts: Firstly, all the images were cropped and segmented. Secondly, a 3D model was built utilizing the processed images, and the model was deformed by finite element analysis (FEA) and refined by an optimization process. Finally, the affine transformation was applied on the output of FEA to finish PET and MRI registration. Overall, the large-scale deformation of the breast was predicted by FEA with an optimization process. The registration procedures are shown in Figure 3.

#### 2.2.1. Preprocessing

Since the boundaries of PET images were very blurry, CT images and PET images were acquired together and shared the same coordinate. Hence, the CT images were utilized to build the 3D model. The resolutions of both PET and CT images were linearly interpolated to meet the resolution of MRI. The CT images were cropped to contain the ipsilateral breast containing the known lesion. The chest wall and muscle were manually segmented from the breast. Then, the images were segmented into the adipose and fibro-glandular tissues by using a fuzzy-c means algorithm.

#### 2.2.2. Patient-Specific Biomechanical Model

Three-dimensional models of the fatty and fibro-glandular tissues were built based on the segmented CT images, respectively, to be further merged and generate meshes from the built 3D model. Our preliminary results showed that the hexahedron mesh was more efficient than tetrahedral mesh when the number of elements/nodes was the same. The appropriate mesh density was determined by convergence analysis, by making sure that the effect of changing the mesh sizes was no more than 2% in the displacement field simulation result.

Li et al. [17] indicated that skin comprises three layers: the epidermis, dermis, and fatty subcutaneous layers. The deepest layer is the subcutaneous fat layer, which has the least stiffness (around 34 kPa) compared with the other layers of skin, with a thickness over 1.2 mm. For the dermis layer, Young’s modulus is around 88 to 300 kPa and its thickness is around 1 mm. The top layer is the epidermis layer, which has the highest Young’s modulus of approximately 1 MPa, with a thickness of 0.1 mm. Therefore, an extra 1 mm layer of shell mesh was added to the surface of the breast model to model the skin. The adipose tissue, skin, and fibro-glandular tissue were assigned a density of 950 kg/m${}^{3}$, 1000 kg/m${}^{3}$, and 1020 kg/m${}^{3}$ [18], as shown in Table 2. The model was assigned a homogeneous isotropic material. The stiffness ratio of the fibro-glandular and fatty tissues was chosen based on published data. The Young’s modulus of the fibro-glandular tissue was modeled as 7.5 higher than that of the fatty tissue [13]. According to previous studies, breasts are considered incompressible [19,20,21,22]. As breast tissue exhibits nearly incompressible, nonlinear, and hyperelastic behavior, the neo-Hookean model was adopted to describe the stress and strain relationship. The strain energy density function was given by

(1) $$\psi =\frac{\mu }{2}\left({\bar{I}}_{1}-3\right)+\frac{K}{2}{\left(J-1\right)}^{2},$$

where ${\bar{I}}_{1}=\left(J-2/3\right)$ is the first invariant of the isochoric part, J=det(F) is the determinant of the deformation gradient, *F*, μ, is the initial small strain shear modulus, and *K* is the initial bulk modulus. Initial shear modulus and bulk modulus can be calculated based on the Young’s modulus, *E*, and Poisson’s ratio, υ, through the relationships of μ=E/(2(1+υ)) and K=E/(3(1−2υ)). The =Poisson’s ratio used in this study was 0.49, according to previous studies, by assuming that the breasts were incompressible [18,19,20,21].

Since all images (CT, PET, and MRI) of the breasts were obtained under a gravity-loading environment, the originally built model was deformed under gravity. Identification of the reference state (load-free state) can make the prediction of deformation more reliable [21]. In this study, the reference state was estimated by calculating the initial stress. The physical behavior was also described by the boundary conditions, in which the surface connected to the chest wall was fixed in the anteroposterior direction to simulate the fixation at the chest wall.

### 2.3. Optimization and Registration

The adipose and fibro-glandular tissues and the skin obey the neo-Hookean constitutive relationship. Landmarks were recognized in the PET/CT images, which were the nipples in the CT and MR images. The nipple displacement from a supine to prone position was used as the standard to optimize the model. A rigid pre-registration was conducted on CT and MRI images to calculate the maximum nipple displacement.

**Figure 4:**
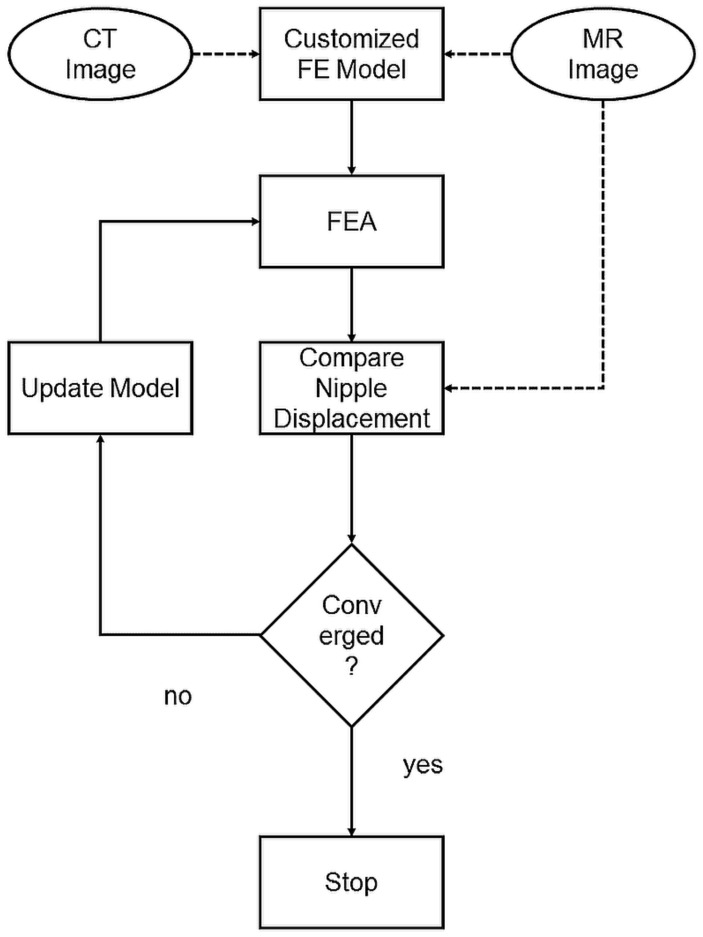
Optimization process: Customized FE model built with CT images. The predicted nipple displacement was compared with real displacement in MR images. The model was then updated by refining material properties until nipple displacements converged.

An optimization procedure was conducted after each finite element analysis, where the nipple displacement was compared with the maximum displacement. The optimization was performed by MATLAB (MATLAB2015, The MathWorks Inc., Natick, MA, USA). The Young’s modulus of the fatty and fibro-glandular tissues was determined through a grid search to minimize the differences in nipple displacement. The optimization process is shown in Figure 4. The optimization stops until the difference between the simulated nipple displacement and the maximum nipple displacement is within 1 mm. Equation (2) is the stopping condition of the Young’s modulus. After completing the optimization, displacement of each node within the 3D model was extracted and interpolated to the pixel size of the PET image to acquire a deformed PET image.

(2) $$argminV_{max}-V_{n}\;\mathrm{subject}\;\mathrm{to}\text{:}\;E\in \left[lb,ub\right]$$

where $V_{max}$ is the maximum displacement of the nipple, $V_{n}$ represents the deformation vector of the nipple during optimization, *E* is the Young’s modulus of adipose tissue, lb is the lower bound constraint for *E*, and ub is the upper limit constraint for *E*. The boundary condition is assigned according to Equation (3).

(3) 0.5 kPa<E<500 kPa.

Affine transformation was applied to further refine the output of FEA and the optimization procedure, as it does not overly depend on image intensity. The registration was directly applied to the deformed PET image and MR images. The landmarks used to calculate the transformation were three manually selected points on the contours of the chest wall based on the shape of the chest wall. The transformation was then applied to the PET images to generate new PET images. The new images were overlapped with MR images to complete the registration.

## 3. Results

### 3.1. Model Performance

**Figure 5:**
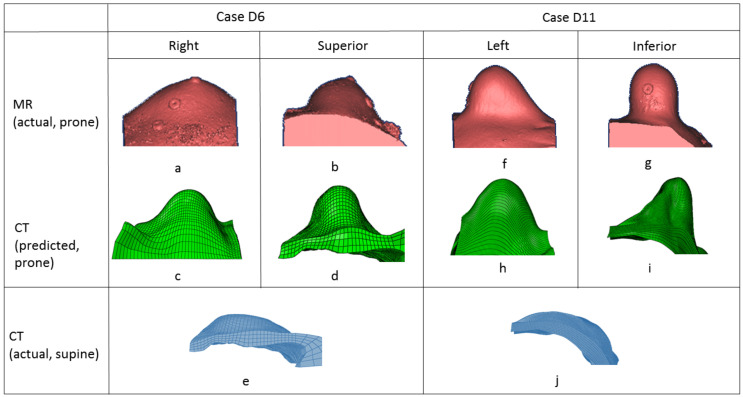
Comparison of predicted breast deformation (**second row**) and real breast deformation (**first row**) in prone position. Original breast model in supine position (**third row**).

Two randomly chosen pairs (case US6 and case US11) of the predicted and original breast models in the prone position are shown in Figure 5. The pink-colored model is built from MR images in the prone position and the green-colored model is the predicted one from CT. The blue-colored one is from the CT images in the original supine position. The differences between the CT images in the supine position (e), (j) and the MR images in the prone position (b), (l) are extremely large, which means that simulation of the deformation is challenging. The predicted model (c, d), (h, i) and the original model (a, b), (f, g) are compared in the superior–inferior and lateral–medial directions. These 3D models showed that the developed model could predict a large deformation of the breast.

### 3.2. Registration Error

The 3D target registration error (TRE) was used in this study to evaluate the registration method. The TRE was defined as the Euclidean distance between the centroid of the landmark in the MR image ($C_{MR}$) and PET image ($C_{PET}$) as shown in Equation (4). We chose the lesion in both PET and MR images as the landmark. The graphic centroids of the landmarks in both the predicted PET and MR images were identified.

(4) $$TRE=\left|\right|C_{MR}-C_{PET}\left|\right|.$$

**Figure 6:**
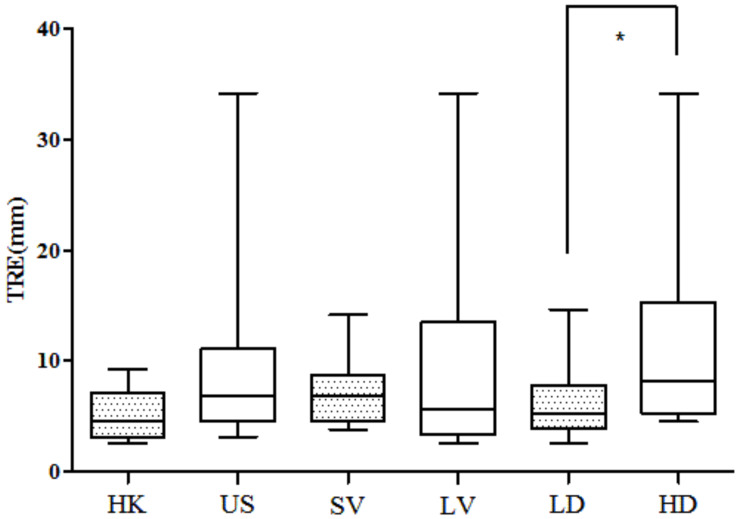
The 3D TRE compared between HK and US groups, 3D TRE compared between small-volume (SV) group and large-volume (LV) group. The 3D TRE compared between low-density (LD) group and high-density (HD) group. No significant difference is found in the first two comparisons. Asterisk means significant difference.

The target registration error of the lesion was calculated in three-dimensional coordinates, rather than within a 2D plane. The value of 3D TRE is shown in Table 3, with a mean value of 8.05 mm. The TRE decreased compared to the affine transformation based on similarity conditions. To assess the effect of density on registration, the 25 cases were divided into a low-density group (LD) and high-density group (HD) with a threshold of 25%. The median value of volume was used as the threshold that divided all the cases into a small-volume group (SV) and large-volume group (LV). Finally, the TRE was also averaged across all cases from each institution in the United States (US) vs. Hong Kong (HK). These values are all plotted in Figure 6. We can see that the TRE value in the LD group is significantly smaller than the HD group (unpaired *t*-test, *p* < 0.05). However, there is no significant difference between SV and LV groups. The TRE of the US cases ranges from 3.11 mm to 34.18 mm, with a mean value of 9.14 ± 7.41 mm. The TRE value of the HK cases is 5.17 ± 2.34 mm. The registration error is not significantly different between the US and HK cases.

**Figure 7:**
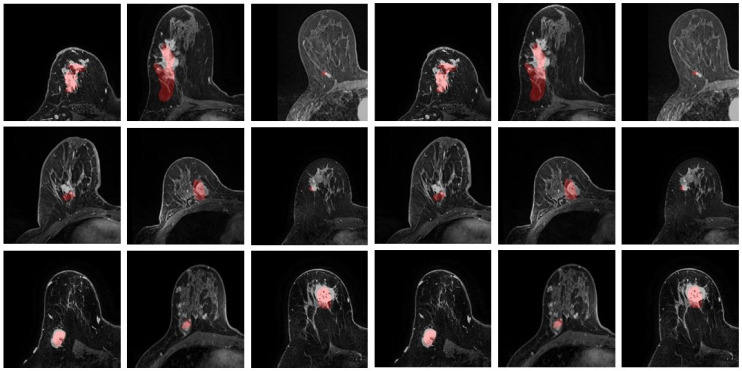
Predicted lesion location. The lesions from PET are marked in red.

**Figure 8:**
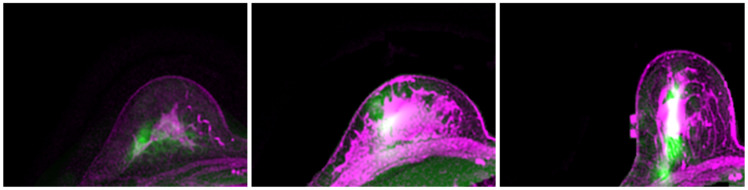
Registered PET (green) and MR (purple) images.

The predicted location of the lesion compared to the lesion in the MR images is shown in Figure 7. The lesion in the PET images is marked in red. Three randomly chosen registered images are shown in Figure 8, with green representing deformed PET images and purple representing the MR images. It can be observed that with the deformed PET images, the location of the lesion can be accurately predicted. The registered PET/MR images provide detailed structural information as well functional information on the lesion region.

### 3.3. Correlation Analysis

Six image features were extracted from the PET/CT images, including patient weight, lesion size, nipple movement displacement, location of the lesion, image density, and breast volume. The location of the lesion is defined as a ratio of the distance of the lesion to the chest wall divided by the distance of the farthest vertices to the chest wall. When the lesion is near the chest wall, this value is close to 0. When the lesion is near the surface of the breast, this value is close to 1.

**Figure 9:**
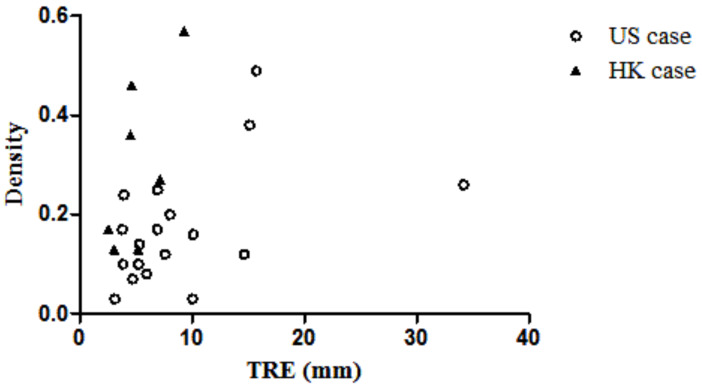
Scatter plot of TRE vs. density of the breast.

The six image features extracted from the PET/CT images and the TRE were analyzed to identify their relationships. However, no correlation was found when analyzing the HK cases and the US cases together. While the density and TRE of the HK cases have a correlation of 0.67, due to the small sample size of HK cases, this correlation is not significant. The density of the US cases was found to be correlated with the TRE value (R = 0.47, *p* < 0.05). However, other features do not show any significant correlation with TRE. The TRE and corresponding density are plotted in Figure 9.

### 3.4. Mechanical Properties

To evaluate our patient-specific model, we also compared the predicted mechanical properties of our method with some relevant studies [23,24,25,26]. An inverse FEA and optimization process were combined to predict the elastic properties of the adipose and fibro-glandular tissues. This method could predict the mechanical properties, which may not necessarily be a real value, but provide an overall trend. As shown in Table 4, the predicted moduli of the breast tissue in the American sample are 0.94 ± 0.37 kPa for the adipose tissue and 7.08 ± 2.81 kPa for the fibro-glandular tissue. They are 2.70 ± 1.84 kPa for the HK adipose tissue and 20.25 ± 13.80 kPa for the HK fibro-glandular tissue. The Young’s modulus of fat tissue for all the cases is 1.44 ± 1.27 kPa, and that of fibro-glandular tissue is 10.77 ± 9.50 kPa. The results show that the Young’s modulus of the US cases is smaller than the HK cases. A comparison of the predicted moduli with the published literature [23,24,25,26] is shown in Table 5. The range of the moduli predicted by our study is comparable with previous studies.

## 4. Discussion

We proposed a patient-specific biomechanical model for PET/CT and MRI registration. The method was evaluated on 25 patients from America and Hong Kong. Specifically, the breast images collected from the American women show significantly lower breast density than those of the Hong Kong sample (p<0.05). This is the first study, to our knowledge, where subjects from two countries with very different racial compositions have been used to evaluate a biomechanical model. The versatility of the model is therefore validated.

To address the non-rigid large deformation of the breast from a supine to prone position, we proposed to adopt the FEA to simulate the deformation of the breast. Considering the mechanical property variance in different patients, we proposed an optimization procedure to build a patient-specific mechanical model, where the mechanical property of each patient is automatically optimized to find the most optimized model for each patient. Then, the deformed PET images were further refined by affine transformation to reduce the residual error. As a result, our method outperformed the affine registration based on image similarity by a large margin.

The model performance has been tested on the two datasets independently. By analyzing the collected breast image datasets from HK and the US, it can be observed that the breast densities of US cases were significantly lower than the HK cases, while the volumes had no significant differences. The TRE of the American sample was 77% greater than that of the Hong Kong sample, but the difference was not significant. The reason might be that, for the US samples, the breast sags towards the abdomen instead of laterally due to gravity, which makes the modeling difficult. There were two statistically significant trends: The density is correlated to the TRE in the US group, and the TRE in the low-density group is smaller than the high-density group. This indicates that the developed biomechanical model in this study can more accurately predict the deformation of breasts with less density. The small registration error for both the American and Hong Kong samples indicated that our proposed biomechanical model of the breast can accurately predict breast deformation regardless of the breast size and this registration method can be widely applied.

To evaluate the efficiency of our proposed patient-specific model, besides the TRE measurement, the predicted mechanical properties of our method were also compared with previous studies. The Young’s moduli of fibro-glandular and adipose tissue predicted in this study are comparable with the values of previous works, as shown in Table 5, which shows the robustness of our method. In the future, we would like to extend our method by adopting deep neural networks [27] for a further refined registration.

## 5. Conclusions

We proposed a patient-specific biomechanical model-based registration method for PET and MR breast images. The method was evaluated by using two independent sets of images from American and Hong Kong institutions. In conclusion, this developed model can accurately predict the deformation of the breast from supine to prone positions for both Asian and Caucasian samples.

## Figures and Tables

**Table 1:** Comparison of breast volume for US and HK samples.

	US Sample	HK Sample
Median of breast volume (mL)	990	762
Median of breast volumetric density	0.15	0.27

**Table 2:** Initial material properties of breast tissues.

	Density (kg/m${}^{3}$)	Young’s Modulus (kPa)	Poisson’s Ratio
Adipose	950	250	0.49
Fibro-glandular	1020	1875	0.49
Skin	1000	1000	0.49

**Table 3:** Comparison between our method and affine registration.

Method	Mean	Std.	Max.
Affine registration	36.68	17.1	61.5
This method	8.05	6.6	34.18

**Table 4:** Predicted Young’s modulus.

Young’s Modulus (kPa)	US	HK	ALL
Fibro-glandular	7.08 ± 2.81	20.25 ± 13.80	10.77 ± 9.50
Adipose	0.94 ± 0.37	2.70 ± 1.84	1.44 ± 1.27

**Table 5:** Comparison of the predicted Young’s modulus.

Young’s Modulus (kPa)	Fibro-Glandular	Adipose
Roose et al. [23]	1.7–500	1.7–500
Tanner et al. [24]	1–20	1
Gefen and Dilmoney [25]	7.5–66	0.5–25
Dufaye et al. [26]	0.5–10	0.1–2
Ours	1.2–20.2	0.2–3.7

## Data Availability

The clinical and MRI data are not publicly available for patient privacy protection purposes.
